# A Post-Surgical Stereovision Surprise in an Adult With an Exotropia Since Infancy Previously Managed, at Two Years With Surgery

**DOI:** 10.22599/bioj.174

**Published:** 2021-06-16

**Authors:** Revelle A. Littlewood, Martin Rhodes, John Burke

**Affiliations:** 1 Sheffield Teaching Hospitals NHSFT, GB

**Keywords:** infant onset exotropia, fusion, strabismus surgery, cosmetic, functional

## Abstract

**Aim:**

To describe an unexpected sensory outcome in an adult male who is seeking ocular re-alignment for a psychosocially symptomatic large non-specific exotropia with suppression. The primary diagnosis was infant onset exodeviation of unclear diagnosis, was managed with bilateral strabismus surgery at two years of age, little memory of follow-up.

**Result:**

Measurable binocular single vision (BSV) was demonstrable following surgery at 17 years of age, albeit slowly between two weeks and six months postoperatively and subsequently enhanced. His newly acquired sub-optimal BSV led to symptomatic occupation-associated asthenopia. Following two subsequent operations over a 15-year period, he has stable, symptom-free ocular realignment within three prism diopters of orthophoria and performing tasks that require extended periods of near-vision activity.

**Conclusion:**

Delayed high levels of stereovision were unexpectedly achieved in an adult with infant onset exotropia with pre-operative sensory suppression that was surgically aligned to near orthophoria. The re-establishment of BSV in such a clinical scenario has to attain a level that is robust enough to meet an individual’s social and occupational needs.

## Introduction

The lack of a reliable history is all too common in childhood onset strabismus cases that re-present itself later in life to the adult motility service. This can present management and prognostic challenges in patients with infant onset exotropia, where the spectrum of sensorimotor outcomes following strabismus surgery in adulthood are not well reported, when compared to patients presenting with consecutive exotropia (Lal & Holmes 2002; Morris, Scott & Dickey 1993) or post-surgery deteriorated intermittent exotropia (Baker 2008; Lim et al. 2008). We describe the 20-year management of a male with a surgically treated infant onset exodeviation who re-presented in adulthood with a psychosocially symptomatic exotropia. The latter was surgically managed. He subsequently benefited from protracted ongoing care including two further procedures for a decompensating exotropia associated with a maldeveloped binocular single vision system that impacted him socially and professionally.

## Case Report

A 16-year-old male presented to the adult motility clinic with a psychosocially symptomatic constant exotropia, which had been troubling him, especially in photographs, for some years. He never experienced diplopia and requested surgical management of his strabismus. His past ophthalmic history included bilateral lateral rectus recessions for a left divergent squint age two-years. Hospital records of his previous strabismus surgery were not available. The duration of post-operative follow-up was thought to be brief but was otherwise unknown. His parents recall the exodeviation to be present in the first year of life but did not clearly recall whether it was intermittent or constant. He was otherwise fit and well with no medical history of note.

**Figure 1:**
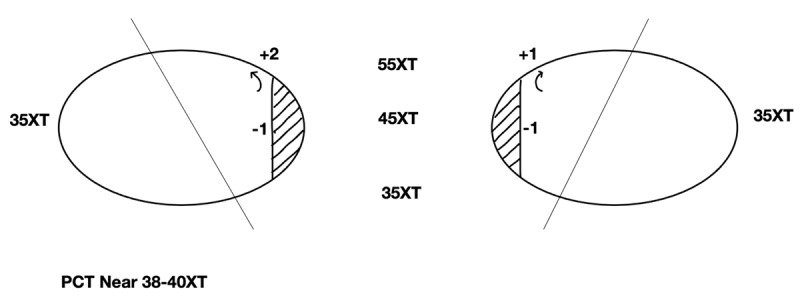
Ocular motility assessment aged 17 years (pre-operation 2), with 6m distance measurements.

Corrected visual acuity was 0.0 Log MAR in either eye (OD –5.75/+1.50×085, OS –4.50/+0.50×085) with a stable refractive error during follow-up in our department. Slit lamp examination revealed conjunctival scarring over the right lateral rectus. An alternate prism and cover test (APCT) measured 45 prism diopters (PD) exotropia at six meters and 40 PD exotropia for near viewing. Ocular motility testing revealed bilateral minor adduction deficits (***Figure 1***). Synoptophore measurements were negative for simultaneous perception while the Worth 4 Dot test (W4D) found alternating suppression at both near and distance. Peripheral testing was not performed.

The patient underwent surgery, namely a bilateral 4 mm medial rectus muscle resection and right lateral rectus re-recession of 3 mms’. The lateral rectus muscle was found at 13 mm from the limbus and further recessed to 16 mm from the limbus (Operation 2). As no BSV potential had been demonstrated, simultaneous bilateral inferior oblique muscle weakening was not performed for the mild inferior oblique overaction and asymptomatic V-pattern.

On the first day post-operatively, he was pleased with the outcome of reduced angle 4 PD exotropia for both near and distance. At the second post-operative visit at two-weeks, he had further improved and was able to momentarily control to an 8 PD exophoria for near and distance in primary position but was not able to demonstrate stereopsis, motor fusion or simultaneous perception.

**Figure 2:**
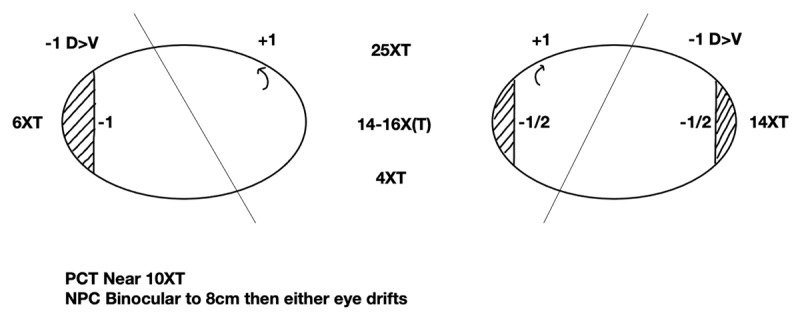
Ocular motility assessment aged 17 years (post-operation 2/pre-operation 3), with 6m distance measurements.

Six months post operatively, at the third post-operative visit, the patient remained pleased with the outcome. The angle in primary position was 14 PD exophoria at 6m and 10 PD exophoria for near (***Figure 2***). The patient could converge to 8 cm then either eye diverged without diplopia. His prism fusion range at near and distance was 4 PD base-out (BO) to 10 PD base-in (BI) with 55’’ (arc/second) stereopsis using the Frisby Stereo test.

**Figure 3:**
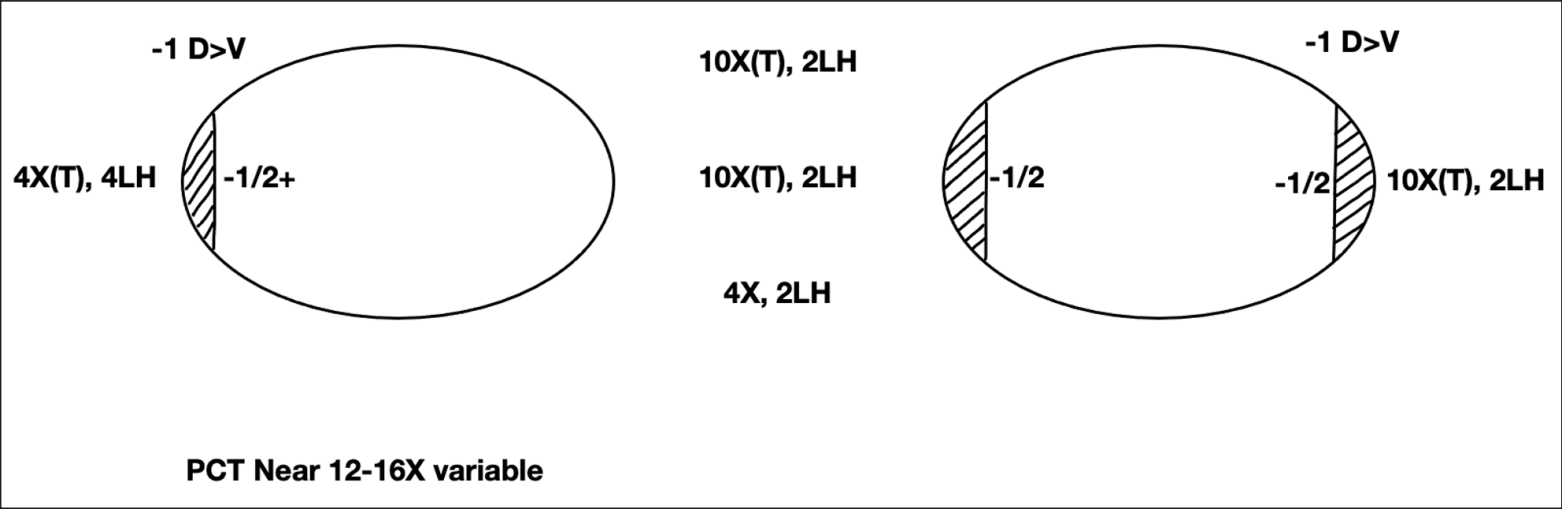
Ocular motility assessment aged 32 years (pre-operation 4), with 6m distance measurements.

Over the next 15 years, he had two more surgeries (Operations 3 and 4) for recurring asthenopia symptoms (headaches and sometimes blurred vision and/or diplopia) related to sustained near-vision tasks whilst working long hours in the software industry. At age 28 years, these included bilateral 10 mm inferior oblique muscle recessions and left lateral rectus re-recession from 12.5 mm to 16 mms’ from the limbus (Operation 3) for a decompensating primary position exophoria associated with persistent symptom-producing mild bilateral overacting inferior oblique muscles and with an increase in the magnitude of the up-gaze exotropia. The rationale for symmetrical additional inferior oblique recessions was to collapse the pattern, thereby reducing the larger incomitant up-gaze exodeviation that could exceed his fusional convergence amplitudes. Following Operation 3 he could converge to 12–14 cm before the left eye diverged. At that time, a fusion range at 6 meters could not be measured as the patient decompensated on introduction of a prism. His asthenopia symptoms initially reduced, only to recur circa three years later. At age 32, he decided to proceed with further surgery, namely a 4 mm right medal rectus re-resection (Operation 4) for recurrent symptomatic decompensating exophoria during the course of his aforementioned work (***Figure 3***).

**Figure 4:**
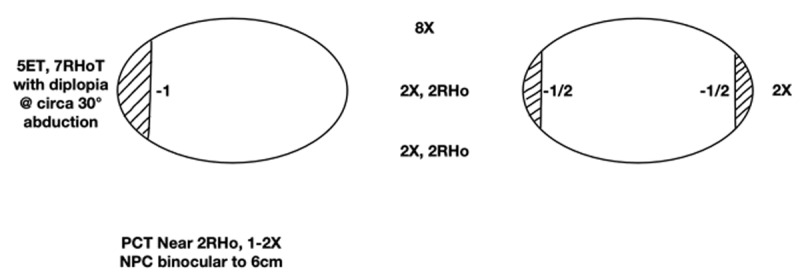
Ocular motility assessment aged 36 years (4 years post-operation 4), with 6m distance measurements.

Circa four years following his fourth operation, he remains happy with his sensorimotor status. He has a 2 PD exophoria and 2 PD right hypophoria for both distance and near (***Figure 4***). Bagolini striated glasses showed a BSV response for near and distance in free space. Prism fusion range for near measured 14 PD BO to 4 PD BI. The W4D showed BSV for near and distance alternating at distance with right suppression. His stereopsis measured 55’’ arc (Frisby), and his convergence was binocular to 6 cm at the last recorded assessment. The small and increased post-operative right abduction underaction may have helped with this ongoing stability in his alignment and symptomatic improvement, albeit at the expense of a slight non-symptoms producing increase in right horizontal gaze incomitance (Deacon et al 2014; Graeber & Hunter 2015).

**Figure 5:**
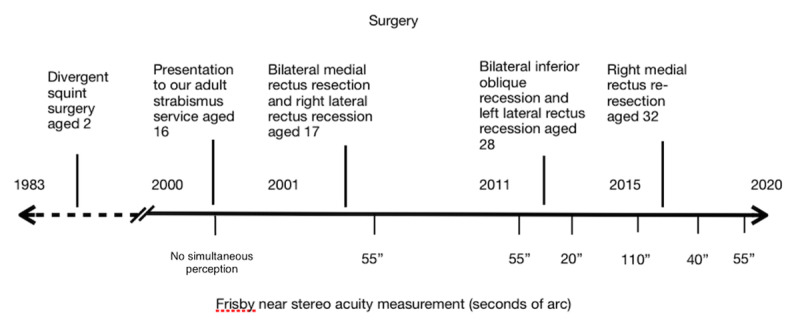
Timeline summary of surgery versus stereoacuity.

Following the three strabismus procedures in adulthood his documented stereopsis improved, the chronology of such stereoacuity and surgical intervention is presented in (***Figure 5***).

## Discussion

Exodeviations account for approximately one quarter of all childhood strabismus cases (Buck et al. 2009), intermittent exotropia (X(T)) being the most common (Govindan et al. 2005). Other causes include exotropia (XT) associated with abnormalities of the central nervous system, convergence insufficiency, congenital XT and XT associated with sensory deprivation or paralytic pathology (Govindan et al. 2005). There are many studies of childhood onset strabismus re-presenting in adulthood, although not specifically infant onset exodeviations. Previous studies highlight the functional improvement that surgical correction to within 15D in primary position can bring (Dickmann et al. 2013; Tomac et al. 2020; Morris, Scott & Dickey 1993).

The term congenital XT is often used interchangeably with infantile XT, encompassing constant XT’s presenting before reaching one-year of age without other ocular pathology in an otherwise healthy child

In our case, we believe the initial differential diagnoses would be limited to infantile XT or intermittent exotropia X(T). Infantile XT in healthy children is far more uncommon than X(T) (Petrunak 2017). Given the close proximity of caregiving tasks for babies and their ability to control at near, it is understandable that the distance exodeviation although present, can be often missed until the child is a little older. In such young patients, it can be difficult to distinguish between these two entities.

Hunter et al. (2001) found that, except for the larger angle observed in congenital XT, distinction between the latter and intermittent exotropia can be difficult in infants presenting within the first 12-months of life, where rates of strabismus associated abnormalities including A- or V-patterns, oblique muscle overaction, nystagmus or dissociated vertical deviation (DVD) are similar between both groups. They raised the question of whether they are simply variable expressions of the same disease where most infantile XTs’ would have an intermittent phase initially while those that are intermittent at presentation can become constant if control is poor.

Maturation of binocular sensory function is almost complete by 18-months of age (Birch 2003), however maturation of binocular vision and susceptibility or vulnerability to deterioration are two separate entities. Developmentally the critical period for susceptibility of stereopsis extends further, beginning at birth, peaking around three to four months of age with continuing susceptibility throughout early childhood to 4.6 years (Fawcett, Wang & Birch 2005). Our case re-presented at age 16, and despite excellent visual acuity in both eyes, synoptophore assessment did not illicit the potential for binocular single-vision potential that subsequently materialised following realignment surgery. This is a clinical scenario that is perhaps recognised in orthoptic practice more frequently than is reported in the literature. Whilst not often performed in patients with a history of infant onset exotropia, a prolonged Prism Adaptation Test (PAT) compliantly performed in a motivated adult may in this situation have shown potential for BSV by allowing more time pre-operatively to re-establish a BSV response than the synoptophore evaluation (Prism adaptation study research group 1990).

Andalib et al. (2015) performed a review of childhood onset strabismus undergoing surgery in adulthood, specifically looking at prognostic factors for sensory outcomes. This more general, non-targeted review included exotropes and esotropes that were not subcategorised further. All had absent stereopsis pre-operatively. Post operatively, it initially appeared that the exotropes had a greater improvement in stereopsis (55.8%) when compared to the esotropes (41.2%). Similarly the non-amblyopic groups (30.4%) showed greater improvement in stereopsis than the amblyopic group (9.1%), however neither of these were statistically significant. The only prognostic factor identified for improvement of stereopsis was the post-operative surgical alignment of orthotropia. Our case met all three positively reported prognostic factors, namely a non-amblyopic exotrope that achieved post-surgery orthophoria.

Koc & Sefi-Yurdakul (2016) looked specifically at visually mature exotropes. Aside from the intermittent nature at presentation, they found other positive predictors of stereoacuity included onset after one year of age and the absence of aniso-acuity. They also believed alphabet patterns, inferior oblique overaction and vertical strabismus, to have a negative predictive role for a positive fine stereoacuity response (better than 60’’ arc). Our patient had two negative predictive factors based on the latter authors findings, namely a historic exotropia onset in the first year of life and a modest V-pattern that was managed with strabismus surgery (Operation 3).

Limited data has been published investigating binocularity in infantile XT, specifically comparing constant infantile XT and X(T). However, Saunders & Trivedi (2008) suggest that at least gross binocularity is achievable in both subgroups, with X(T) more able to achieve measurable stereopsis or even bifoveal fixation than XT. Hunter et al. (2001) retrospectively analysed 13 patients with XT presenting before the age of one year and split their cohort into constant XT and X(T) groups where 92% underwent surgery for constant exotropia or progressive loss of control of X(T). Post-operatively, 27% needed further surgery within the follow up range of 13–158 months. Six of 13 had no BSV, while seven obtained gross BSV, where two of the seven achieved better than 200’’, both being in the X(T) group. Suh et al. (2013) evaluated the outcomes of surgery in 45 children with early onset XT before one year of age, who they split into constant XT and X(T) subgroups. Ten patients achieved Titmus test stereoacuity greater than 60’’ of arc, one in the XT versus nine in the X(T) group. These three studies find that a minority of children with a diagnosis of infant onset exodeviation can develop demonstratable BSV following early childhood surgery with seemingly higher grades of stereoacuity achievable in those young children with intermittent exotropia. We believe that our case did at least experience BSV for some period before and/or after his early childhood surgery during the recognized developmental period of stereovision maturation and vulnerability (Fawcett, Wang & Birch 2005), before decompensating to a constant exotropia with establishment of suppression that was reversed by successful motor re-alignment surgery in adulthood. Our case has maintained measurable stereo-acuity for almost two decades, with, on each occasion of subsequent surgeries (Operations 3 & 4), a slight reduction in stereovision preceding a further surgical intervention and improvement post-operatively (***Figure 5***). Fluctuations of stereo-acuity, even in those who are visually mature can occur and seemingly there is residual neural plasticity (Andalib et al. 2015).

Convergence fusional reserves are linked to control of X(T) (Fu et al. 2015, Hatt et al. 2011). On initial presentation to clinic, our patient had poor convergence and no fusion. However, following Operation 3, he could converge to 12–14 cm before the left eye diverged. At that time, a fusion range at six meters could not be measured as the patient decompensated on introduction of a prism, consistent with his sub optimal BSV development. Following Operation 4, his binocular convergence and fusional convergence reserves at near had further improved.

Although data is limited and mainly retrospective; a minority of patients with infant-onset exotropia, good visual acuities and no demonstratable pre-operative binocular single vision (BSV) potential can develop gross BSV or even measurable stereovision when managed surgically in adulthood. In our patient, this re-established itself gradually, between two weeks and six months post-operatively. The limited literature suggests that the prognosis is consistently better for the infantile onset intermittent exotropia group. Paradoxically, the successful alignment outcome of our particular patient’s psychosocially symptomatic exotropia latterly presented him with secondary asthenopia related BSV challenges largely precipitated by the specific requirements of his career. This necessitated further surgeries to achieve near orthophoria alignment and binocular convergence to 6 cm so as to enable his restored sub-optimal fusion to better meet the demands of his occupation.

## References

[1] Andalib, D, Nabie, R and Poormohammad, B. 2015. Factors affecting improvement of stereopsis following successful surgical correction of childhood strabismus in adults. Strabismus, 23(2): 80–84. DOI: 10.3109/09273972.2015.1025985 26158474

[2] Baker, JD. 2008. Twenty year follow up of surgery for intermittent exotropia. J AAPOS, 12(3): 227–32. DOI: 10.1016/j.jaapos.2008.02.007 18455937

[3] Birch, EE. 2003. Binocular sensory outcomes in accommodative ET. J AAPOS, 7(6): 369–73. DOI: 10.1016/j.jaapos.2003.08.003 14730284

[4] Buck, D, Powell, C, Cumberland, P, Taylor, R, Sloper, J, Tiffin, P, Davis, H, Rahi, J and Clarke, MP. 2009. Presenting features and early management of childhood intermitted exotropia in the UK: Inception cohort study. Br J Ophth, 93(12): 1620. DOI: 10.1136/bjo.2008.152975 19605936

[5] Deacon, BS, Fray, KJ, Grigorian, AP, Qureshi, HM, Spencer, HJ, Lowery, RS and Phillips, PH. 2014. Unilateral strabismus surgery in patients with exotropia results in post-operative lateral incomitance. J AAPOS, 18: 572–575. DOI: 10.1016/j.jaapos.2014.08.010 25498465

[6] Dickmann, A, Sliberti, S, Rebecchi, MT, Aprile, A, Salerni, A, Petroni, S, Parrilla, R, Perrotta, V, Di Nardo, E and Balestrazzi, E. 2013. Improved sensory status and quality-of-life measures in adult patients after strabismus surgery. J AAPOS, 17: 25–28. DOI: 10.1016/j.jaapos.2012.09.017 23352383

[7] Fawcett, SL, Wang, Y and Birch, EE. 2005. The critical period for susceptibility of human stereopsis. Invest Ophthalmol Vis Sci, 46: 521–525. DOI: 10.1167/iovs.04-0175 15671277

[8] Fu, T, Levin, M, Su, Q, Li, D and Li, J. 2015. Fusional vergence detected by prism bar and synoptophore in Chinese childhood intermittent exotropia. J Ophthalmol, 1–6. DOI: 10.1155/2015/987048 PMC4411439 25954512

[9] Govindan, M, Mohney, BG, Diehl, NN and Burke, JP. 2005. Incidence and types of childhood exotropia: A population-based study. Ophthalmology, 122: 104–8. DOI: 10.1016/j.ophtha.2004.07.033 15629828

[10] Graeber, CP and Hunter, DG. 2015. Changes in lateral comitance after asymmetric horizontal strabismus surgery. JAMA Ophthalmol, 133: 1241–6. DOI: 10.1001/jamaophthalmol.2015.2721 26291652

[11] Hatt, SR, Leske, DA, Mohney, BG, Brodsky, MC and Holmes, JM. 2011. Fusional convergence in childhood intermittent exotropia. Am J Ophthalmol, 152(2): 314–9. DOI: 10.1016/j.ajo.2011.01.042 21621744 PMC3170040

[12] Hunter, DH, Barrett-Kelly, J, Buffenn, AN and Ellis, FJ. 2001. Long term outcome of uncomplicated infantile exotropia. J AAPOS, 5(6): 352–56. DOI: 10.1067/mpa.2001.120175 11753254

[13] Koc, F and Sefi-Yurdakul, N. 2016. Predictors of stereoacuity outcome in visually mature subjects with exotropia. Eye (Lond), 30: 264–269. DOI: 10.1038/eye.2015.241 26584792 PMC4763128

[14] Lal, G and Holmes, JM. 2002. Postoperative stereoacuity following realignment for chronic acquired strabismus in adults. J AAPOS, 6: 233–237. DOI: 10.1067/mpa.2002.123399 12185349

[15] Morris, RJ, Scott, WE and Dickey, CF. 1993. Fusion after surgical alignment of longstanding strabismus in adults. Ophthalmology, 100(1): 135–8. DOI: 10.1016/S0161-6420(93)31703-3 8433818

[16] Petrunak, JL. 2017. Everyday exotropia: learning from the littlest. Am Orthop Journal, 67: 52–60. DOI: 10.1080/0065955X.2017.12023633 28904215

[17] Prism Adaptation study research group. 1990. Efficacy of prism adaptation in the surgical management of acquired esotropia. Arch Ophthalmol, 108(9): 1248–56. DOI: 10.1001/archopht.1990.01070110064026 2100986

[18] Saunders, RA and Trivedi, RH. 2008. Sensory results after lateral rectus muscle recession for intermittent exotropia operated before two years of age. J AAPOS, 12: 132–135. DOI: 10.1016/j.jaapos.2007.08.011 18083585

[19] Suh, SY, Kim, MJ, Choi, J and Kim, SJ. 2013. Outcomes of surgery in children with early-onset exotropia. Eye (Lond), 27: 836–40. DOI: 10.1038/eye.2013.75 23619215 PMC3709395

[20] Tomac, S, Uyar, E, Akin, T, Mutlu, FM and Altınsoy, HI. 2020. Late surgical correction of longstanding constant strabismus in adults: Is fusion possible in all successfully aligned patients? J Binocul Vis Ocul Motil, 70: 109–114. DOI: 10.1080/2576117X.2020.1787017 32673179

